# An *Sp185/333 *gene cluster from the purple sea urchin and putative microsatellite-mediated gene diversification

**DOI:** 10.1186/1471-2164-11-575

**Published:** 2010-10-18

**Authors:** Chase A Miller, Katherine M Buckley, Rebecca L Easley, L Courtney Smith

**Affiliations:** 1 Genomics and Bioinformatics Program, Department of Biochemistry, School of Medicine, The George Washington University, Washington DC, 20037, USA; 2 Department of Biological Sciences, George Washington University, Washington, DC, 20052, USA; 3 Department of Biology, Boston College, Boston, MA, USA; 4 Department of Immunology, Sunnybrook Research Institute, University of Toronto, Toronto, ON, Canada; 5 TECHLAB Inc, Blacksburg, VA, USA

## Abstract

**Background:**

The immune system of the purple sea urchin, *Strongylocentrotus purpuratus*, is complex and sophisticated. An important component of sea urchin immunity is the *Sp185/333 *gene family, which is significantly upregulated in immunologically challenged animals. The *Sp185/333 *genes are less than 2 kb with two exons and are members of a large diverse family composed of greater than 40 genes. The *S. purpuratus *genome assembly, however, contains only six *Sp185/333 *genes. This underrepresentation could be due to the difficulties that large gene families present in shotgun assembly, where multiple similar genes can be collapsed into a single consensus gene.

**Results:**

To understand the genomic organization of the *Sp185/333 *gene family, a BAC insert containing *Sp185/333 *genes was assembled, with careful attention to avoiding artifacts resulting from collapse or artificial duplication/expansion of very similar genes. Twelve candidate BAC assemblies were generated with varying parameters and the optimal assembly was identified by PCR, restriction digests, and subclone sequencing. The validated assembly contained six *Sp185/333 *genes that were clustered in a 34 kb region at one end of the BAC with five of the six genes tightly clustered within 20 kb. The *Sp185/333 *genes in this cluster were no more similar to each other than to previously sequenced *Sp185/333 *genes isolated from three different animals. This was unexpected given their proximity and putative effects of gene homogenization in closely linked, similar genes. All six genes displayed significant similarity including both 5' and 3' flanking regions, which were bounded by microsatellites. Three of the *Sp185/333 *genes and their flanking regions were tandemly duplicated such that each repeated segment consisted of a gene plus 0.7 kb 5' and 2.4 kb 3' of the gene (4.5 kb total). Both edges of the segmental duplications were bounded by different microsatellites.

**Conclusions:**

The high sequence similarity of the *Sp185/333 *genes and flanking regions, suggests that the microsatellites may promote genomic instability and are involved with gene duplication and/or gene conversion and the extraordinary sequence diversity of this family.

## Background

Invertebrate immune systems are marked by an array of complex and sophisticated mechanisms for recognizing and responding to microbes [1-4]. A few systems that highlight this complexity are reshaping the paradigm that invertebrate immune systems were thought to be simple. The genes that encode fibrinogen-related proteins (FRePs) in the freshwater snail *Biomphalaria glabrata *diversify through somatic diversification and point mutation of a small gene set [5]. Arthropod *DSCAM *genes employ extensive alternative splicing to generate thousands of unique mRNAs [6-8] that encode proteins involved in phagocytosis by hemocytes [9] and may bind specifically to the infecting pathogen [10]. In higher plants, a variety of classes of *R *genes exhibit disease resistance capabilities, and create and maintain diversity by sequence exchange and recombination (reviewed in [11]). Furthermore, a number of gene families function in immunity in which the mechanisms of diversification have not been investigated, such as the variable region-containing chitin-binding proteins (VCBPs) in protochordates [12-14].

**Figure 1:**
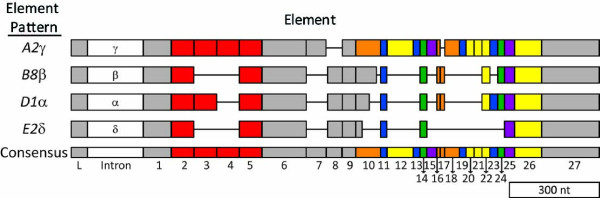
**The *Sp185/333 *genes on 7096 have four different element patterns**. The genes are aligned according to the repeat-based alignment [15]. The genes have two exons and a single intron that is shown as a white box (not to scale). The first exon encodes the leader (L). The Greek letters indicate the intron type based on sequence analysis [15]. The second exon has large gaps (horizontal lines) inserted to optimize the alignment, which define blocks of sequence called elements (gray and colored boxes). The consensus of all possible elements is shown at the bottom. Variations in the presence or absence of elements defines element patterns (*A2*γ, *B8*β, *D1*α, and *E2*δ, which are abbreviated according to [15]). Elements that correlate with each of the six types of repeats are shown in different colors (type 1 in red; type 2 in blue; type 3 in green; type 4 in yellow; type 5 in purple; type 6 in orange; [15]). The figure is modified from [15].

The diverse, immune related gene family called *185/333*, has been identified in several species of sea urchins [15-19]; D.A. Raftos, M. Roth, N.M. Dheilly, unpublished; K.M. Buckley, L.C. Smith, unpublished). The best understood of these homologues is the *Sp185/333 *gene family in the purple sea urchin, *Strongylocentrotus purpuratus*. *Sp185/333 *genes appear to have an immunological role and are highly expressed in coelomocytes responding to challenge with whole bacteria [17,20] lipopolysaccharide [17,18], β-1,3-glucan, double-stranded RNA [18], and peptidoglycan [21]. Sea urchin larvae express *Sp185/333 *in blastocoelar cells when grown with marine microbes [16]. Consistent with an immune function, the *Sp185/333 *gene family is extraordinarily diverse. Alignment of the *Sp185/333 *sequences defines blocks of shared sequence known as *elements *based on the locations of large gaps (Figure 1) [17]. The variable presence and absence of these elements in different genes defines *element patterns*. Analysis of the evolutionary histories of these elements suggests that the extant genes are the result of recent diversification through frequent recombination such that the genes contain a mosaic distribution of element sequences and appear to be hybrids of other extant genes [22]. The gene family is estimated to contain around 50 genes based on three lines of evidence: 1) statistical analysis of the unique *Sp185/333 *genes given the total number cloned from three individual animals, 2) quantitative PCR (qPCR) analysis of alleles in sea urchin genomic DNA (gDNA), and 3) estimates from BAC library screens [16,19,22]. PCR amplification of intergenic regions suggests that at least some of the genes are closely linked and are positioned in various orientations [15]. The *S. purpuratus *genome assembly (v2.5), however, contains only six *Sp185/333 *genes on two scaffolds [23].

Shotgun sequence assembly is the standard method for quick and efficient assembly of BACs and whole genomes but there are problems in correctly assembling regions with repetitive elements. The most common type of gap in 'finished' genomes are unresolved heterochromatin regions, which are mainly composed of repetitive elements [24,25]. Much effort has gone into improving the assembly of these types of regions and some progress has been made with assembling transposons using specific transposon-based approaches [26]. However, these methods fail when applied to the assembly of other repetitive elements. A detailed study of mis-assembled segmental duplications in the 'finished' human genome shows that shotgun strategies consistently mis-assemble segmental duplications that are at least 15 kb and share at least 97% identity [27]. Although shotgun assembly is extremely flexible and powerful, it can be modified to improve results especially when a specifically defined goal is included in the approach [25,28,29]. The significant underrepresentation of *Sp185/333 *genes in the sea urchin genome compared to our estimates of the gene family size may stem from two possible sources. First, the numbers of trace sequences with *Sp185/333 *sequence that were used to assemble the genome are fewer than expected, and may result from gene deletions from BAC inserts during growth of the cultures. This possibility will be tested in the future. Second, the genes may be incorrectly assembled in the genome because repetitive sequences are commonly mis-assembled and are often collapsed onto a single genomic location [24,25,27]. This second possibility is addressed below.

We report here the first follow-up to the problem of assembling the *Sp185/333 *genes, and show how the shortcomings of shotgun assembly for these genes could be overcome by focusing on a single BAC insert, an easier task for a repeat-riddled region. We generated multiple candidate BAC assemblies with varying parameters to account for potential gene collapse or artificial duplication/expansion, and experimentally validated the assemblies to identify the optimal sequence. We present a unique perspective on sequence assembly and validation, particularly the need to adjust the assembly parameters locally, rather than using global parameters for the entire genome. This is the first report of a small cluster of six *Sp185/333 *genes in a 34 kb region located at one end of a 117 kb BAC insert. The gene structure is consistent with that of previously characterized *Sp185/333 *genes; the coding region is contained within two exons, the second of which includes the mosaic pattern of elements [15]. All six genes are flanked on both sides by GA microsatellites and four of the genes have a GAT microsatellite in the 5' flank. There is no correlation between linkage and sequence similarity, as the six genes on the BAC are no more similar to each other than to 121 unique genes that have been cloned and sequenced from three different animals [15]. The flanking regions of the genes that extend to the microsatellites exhibit significant sequence similarity. Three of the *Sp185/333 *genes are tandemly duplicated including their flanking regions and each repeated segment is delineated by microsatellites. The assembly of this region had to be validated by cloning and sequencing. The very high sequence similarity of the *Sp185/333 *genes, the flanking regions, and the positions of the flanking microsatellites may promote genomic instability and increase the rate of gene duplication of this family and/or perhaps block homogenization resulting from gene conversion, thereby contributing to its extraordinary diversity.

## Methods

### BAC library screening

Two arrayed BAC libraries (Sp BAC genomic and Sp small BAC; http://www.spbase.org/SpBase/resources/index.php) were screened for clones with *Sp185/333 *sequences [15]. The libraries differed in average insert sizes (Sp BAC genomic library inserts were ~140 kb, 25× genome coverage; Sp small BAC library inserts were ~50 - 80 kb, 6.25× genome coverage) [30]. The libraries were screened with riboprobes synthesized from combinations of templates chosen from three *Sp185/333 *gene clones that included all known elements (10-010 [GenBank:EF607629; element pattern *G2*γ], 10-022 [GenBank:EF607640; element pattern *D1*α], and 2-095 [GenBank:EF607756; element pattern *E2*δ]) [15]. The Sp small BAC library was screened as previously described for the Sp BAC genomic library [15]. Riboprobe synthesis and filter hybridization were performed as described in [31]. BAC clones with *Sp185/333 *sequence were obtained from Eric Davidson and Andrew Cameron at the California Institute of Technology.

### BAC insert isolation and PFGE analysis

Bacterial cultures were grown at 37°C with chloramphenical and the BAC plasmids were isolated using the alkaline lysis protocol as described in [15]. The insert was released from the pBACe3.6 vector with *Not*I (New England Biolabs) digestion and analyzed by pulsed-field gel electrophoresis (PFGE) with 1% Pulsed Field Certified Agarose (Bio-Rad Laboratories) gel in 0.5× TBE at 6 V/cm, and a ramped switch time from 1 to 15 sec over 16 hrs. Gels were stained in 0.5 μg/mL ethidium bromide, destained and imaged under UV light. The MidRange pulsed-field gel (PFG) Marker I (New England Biolabs) was used to generate the standard curve to plot the BAC insert size.

### BAC sequencing

A working draft sequence of BAC clone R3-3033E12 was generated as part of the *S. purpuratus *genome project [GenBank: AC178508.1] [32]. A randomly sheared subclone library was generated from BAC 178508 and end sequencing the subclones was performed at the Baylor College of Medicine (BCM) generating 1,886 traces by Sanger sequencing. Traces were deposited in the NCBI Trace Archive as a BCM center project SRHQ; TI number AC204781.3. The results reported here employ different methods (see following) than those used by the Baylor team to assemble the traces into a BAC insert sequence [GenBank: BK007096], which is hereafter called "7096".

### Assembly

The 7096 sequence was assembled from the traces using the Whole-Genome Shotgun Celera Assembler [33]. Traces were converted into the format required by the Celera Assembler with the tarchive2ca tool, which is part of the A Modular Open Source tool suite http://amos.sourceforge.net/[33]. Assemblies were generated using default parameters, with the exception of varying unitigger error rates that ranged from the default of 1.5% to 0.2% in 0.1% decrements. Hawkeye [34] was used to view the assemblies graphically and to assess sequencing coverage. GenePalette [35] was used to annotate the 7096 assembly.

### Real-time quantitative PCR (qPCR) analysis of Sp185/333 genes on BACs

qPCR was used to estimate the number of *Sp185/333 *genes on the BACs according to [19]. Primers used to amplify the *Sp185/333 *genes were 5'UTF.1 and LR1 (Table 1). The BAC plasmid copy number in each reaction was quantified using primers 17F and 18R (Table 2), which produced a single amplicon from the 7096 insert. Reactions were performed in duplicate under the following conditions: 95°C for 12 min, followed by 40 cycles of 95°C for 15 sec, 59°C for 30 sec, and 72°C for 30 sec. Melt curve analysis confirmed the amplification of a single product. The number of *Sp185/333 *genes on the BAC was determined by dividing the starting quantity of cloned *Sp185/333 *genes by the number of BAC plasmids in each reaction. Standard curves were generated from four 10-fold serial dilutions (10^7 ^- 10^4 ^plasmids/reaction) using two cloned *Sp185/333 *genes (2-095, [GenBank: EF607756]; and a subclone of 7096 generated using primers 17F and 18R; Table 1). Two concentrations of BAC template DNA were used in the reactions.

**Table 1:** Primer locations in the exons or flanking untranslated regions^1^

Primer^2^	Sequence	Strand^3^	Notes
5' UTR.1	YTDTAGCATCGGAGAKACCT^4^	S	5' untranslated region of all genes
F2	AAGMGATTWCAATGAACKRCGAG	S	In the second exon ~500 bp from 5' end of all genes
F5	GGAACYGARGAMGGATCTC	S	In the second exon ~1.4 kb from the 5' end of most genes
F6	GAAGAAGAAACTGATGCTGCC	S	In the second exon ~900 bp from the start codon in all genes.
LR1	ATCRTYGCCATYSTGGCYG	AS	In the first exon ~50 bp from the start codon in all genes.
R5	AAATGGCGGTCCGATGRGTG	AS	In the second exon ~800 bp from the start codon in most genes
R6	GAGAMGAAGAAACTGATGCTGC	AS	In the second exon ~900 bp from the start codon in all genes.
R9	CGACATYTTCACCACYTDAAG	AS	In the second exon ~ 1.5 kb from the 5' end of most genes
3' UTR.1	GTCGCYGAGGTGTAGAATTW	AS	3' end of all genes
3' UTF-1	CGTCATAACCGTACCAAAGAC	S	3' end of some genes

^1^see also [15,19]

^2^F, forward; R, reverse

^3^S = sense; AS = antisense

^4^D = A, G, or T; K = G or T; Y = C or T; R = A or G; M = A or C

**Table 2:** Intergenic primers

Primer^1^	Sequence	Strand^2^	Notes^3^
1R	CGAAGATAAGTAATTGGT	AS	~300 bp 5'of each *D1 *gene
2F	GTTCTGTTTTTAGTACCG	S	RC of 12R, located ~2.2 kb 3' of all *D1 *genes
6F	TTGAGAGCTCGTCACGTG	S	~900 bp 3' of the *D1*-b gene
7F	TGCAATCATTTTACATATTACTGGTT	S	~800 bp 3' of the *A2 *gene
9F	GGGATTACATACCATACCGCA	S	~1 kb 3' of the *B8 *gene
11F	ATCCTTTGAAACAGCCCCTC	S	RC of 10R, located ~2.4 kb 3' of the D1-y gene
13F	TGGGAAATACTGACTGCC	S	RC of 5R, located ~2.7 kb 3' of the *E2 *gene
17F^4^	TTTCCAATGTCCTTATTTACGACTTATA	S	qPCR primer with 18R^1^
21F	AATGTATTCGGCAGCGAGGT	S	~1 kb 3' of the *D1 *genes
5R	GGCAGTCAGTATTTCCCA	AS	RC of 13F, located ~1.1 kb 5' of the *D1*-b gene
8R	AAGCCTGCTGCTCAATCATC	AS	~1.2 kb 5' of the *A2 *gene
10R	GAGGGGCTGTTTCAAAGGAT	AS	RC of 11F, located ~1.1 kb 5' of the *B8 *gene
12R	CGGTACTAAAAACAGAAC	AS	RC of 2F, located ~1 kb 5' of all *D1 *genes
14R	AAGTGGTGGTAGGCTCAGTAGTA	AS	~700 bp 5' of the *E2 *gene
18R^4^	ATGATTCACAGGTTTGTTGCCTC	AS	qPCR primer with 17F^1^

^1^F, forward; R, reverse

^2^S = sense; AS = antisense

^3^RC = reverse complement

^4^These primers amplify a unique region used to quantify the copy number of BAC plasmids in qPCR reactions.

### PCR and cloning

Primers (Table 2) were designed with Primer Premier (Premier Biosoft International, Palo Alto, CA) based on an assembly of 7096 that was generated using 0.9% unitigger error rate. Amplicons of less than 5 kb were produced in reactions with 4 - 20 ng of BAC DNA, 200 nM each primer, 200 μM each dNTP, 1 unit (U) Paq5000 Taq (Stratagene, La Jolla, CA), and 1× company-supplied buffer. Samples were amplified under the following conditions: 3 min at 95°C, followed by 25 cycles of 20 sec at 95°C, 20 sec at 51°C to 59°C and 10 sec to 2.5 min at 72°C, followed by 3 min at 72°C and a 4°C hold. For amplicons longer than 5 kb, each reaction consisted of 0.4 - 2 ng BAC DNA, 200 nM each primer, 400 μM each dNTP, 1 U Takara *LA Taq *(Takara Biosciences, Madison, WI) and 1× company-supplied buffer. Samples were amplified with the following conditions: 3 min at 94°C followed by 30 cycles of 30 sec at 94°C and 5 to 10 min at 51°C to 65°C, followed by 10 min at 72°C, and a 4°C hold.

Amplicons of regions surrounding the *D1 *genes employed PCR reactions with 10 ng of 7096 DNA, 500 nM each primer (1R and 2F; Table 2), 400 μM each dNTP, 1 U PhusionTaq (New England Biolabs, Ipswich, MA), and 1× company-supplied buffer. Samples were amplified as follows: 30 sec at 98°C, 25 cycles of 10 sec at 98°C, 20 sec at 55°C, and 2 min at 72°C, followed by 5 min at 72°C, and a 4°C hold. Amplicons were adenylated by adding 1 U of Fisher Taq (Fisher Scientific, Pittsburgh, PA) to the reaction for 10 min at 72°C to facilitate amplicon cloning into pCR4-XL-TOPO (Invitrogen, Carlsbad, CA). Plasmid DNA (pCR4-XL-TOPO with 7096 fragment inserts) was isolated using the Wizard Plus Miniprep DNA Purification System (Promega, Madison, WI).

### Cycle sequencing

Cycle sequencing reactions consisted of 165 ng of plasmid DNA, 1 μM of each primer, sequencing buffer (267 mM Tris base pH 9.0, 6.7 mM MgCl_2_), 1× dye terminator cycle sequencing (DTCS) Quickstart (Beckman Coulter, Fullerton, CA). Samples were amplified in an iCycler (Bio-Rad Laboratories) with the following conditions: 30 cycles of 20 sec at 96°C, 20 sec at 50°C and 20 sec at 60°C, followed by a hold at 4°C. DNA was precipitated and resuspended in CEQ Sample Loading Solution (Beckman Coulter). Samples were analyzed on a Beckman Coulter CEQ8000 using protocol LFR-a (Beckman Coulter) modified with a 10 second injection duration. Sequences were edited and assembled using Sequencher software (GeneCodes, Ann Arbor, MI).

### Bioinformatics

Sequences were manually aligned using Bioedit [36]. Pairwise diversity was measured by pairwise distance analysis using MEGA v.4 [37] with pairwise deletion of gaps. Dot plots were generated using plotRep [38]. Microsatellites, interspersed repeats, and low complexity DNA sequences were identified by Repeatmasker (http://www.repeatmasker.org). Entropy was calculated as in [15].

## Results

The disagreement between the number of *Sp185/333 *gene models in the *S. purpuratus *genome and our estimates of gene copy number may have resulted from a shortcoming of genome assembly methods, in which regions with similar sequences are artificially collapsed. Consequently, the gene models assembled in the genome may not be sequences of real genes, but rather, may be consensus sequences of multiple genes. Therefore, we analyzed the genomic organization of the *Sp185/333 *genes from the level of a finished BAC sequence. BAC sequences present a simpler computational problem for assembly because there is less sequence to assemble compared to an entire genome from a diploid, outbred animal, and because a BAC is sequence from a single haplotype. This was of particular relevance for the sea urchin, in which genomes have been shown to vary by 4-5% among individuals [39] and the *S. purpuratus *genome assembly is a mosaic of both haplotypes [32].

### BACs with *Sp185/333* sequence

Screens of the large-insert BAC library [30] identified 75 clones that were positive for *Sp185/333 *sequence. Screens of the small-insert BAC library identified 46 positive clones (see [22], reviewed in [16]). Preliminary analysis of the BACs by PCR showed that the *Sp185/333 *genes were positioned in all possible orientations relative to each other and that many BACs had identical patterns of amplicons [15]. PCR, restriction digests and Southern blots of 11 BACs indicated four categories of genes based on the number of shared bands among the groups (data not shown). Two BACs were chosen for sequencing based on different patterns of *Sp185/333 *amplicons and the results for one BAC, 7096, are reported here.

### Assembling the 7096 BAC

An initial sequence for 7096 [GenBank:AC204781] was assembled by the Baylor College of Medicine Human Genome Sequencing Center (BCM-HGSC) using the Phrap assembler [40] as part of the Atlas assembly system [41], with the traces from the randomly sheared subclone library [42]. To validate the sequence assembled by Phrap, the 7096 traces were reassembled with the Celera WGS assembler [43]. The Celera assembler was chosen based on its ability to optimize parameters for contig creation and its relative strengths for correctly assembling repeated regions [43].

Given the high similarity of the *Sp185/333 *genes, it was important to avoid collapsing two similar genes into a single gene and/or creating a non-existent hybrid gene. With this aim in mind, the unitigger error rate, which specifies the threshold of similarity at which two traces are assembled, was adjusted over a range of values. Decreasing the unitigger error rate prevents two or more similar genes from being collapsed into one. However, unitigger error rates that are too low could generate artificial genes from sequencing errors being treated as real single nucleotide polymorphisms (SNPs). In contrast, unitigger error rates that are too high would ignore real SNPs and incorrectly collapse two or more duplicated genes into one. Unitigger error rates ranging from the default of 1.5% plus a range of 1.2% to 0.2% in 0.1% decrements were used to generate 12 assemblies (Table 3).

**Table 3:** Varying assembly parameters affects the length, number of scaffolds, and *Sp185/333 *genes present in different assemblies

				*Sp185/333*	
Assembly^1^	Unitigger rate (%)	Length (nt)^2^	Scaffolds	Genes	Gene Fragments
2	0.2	111,226	5	4	2
3	0.3	120,165	4	5	3
4	0.4	113,898	3	5	1
5	0.5	117,017	2	5	1
6	0.6	110,951	2	4	1
7	0.7	116,484	3	5	1
8	0.8	115,649	2	5	1
9	0.9	117,014	2	5	1
10	1.0	117,098	3	5	1
11	1.1	115,591	3	4	2
12	1.2	116,615	3	4	2
15	1.5	115,620	4	4	2
BCM-HGSC^3^	n/a	119,341	1	6	0

^1^Assemblies 2-15 were generated with the Celera Assembler [33].

^2^excluding gaps

^3^The sequence completed by BCM-HGSC (Genbank: AC204781.3) was assembled using Phrap [40] and Atlas [41].

**Figure 2:**
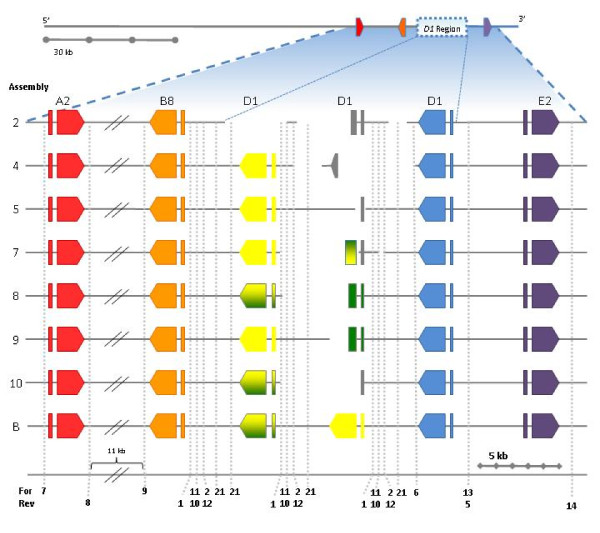
**Varying the unitigger rate affects assembly of the region of 7096 that contains three *D1 *genes**. The sequences assembled using various unitigger rates in the Celera WGS assembler are shown with oriented and ordered scaffolds (see Table 3 for details). The assembly generated by Baylor (Genbank:AC204781.3) is shown at the bottom (B). Sequence differences and scaffold fragmentation among the assemblies is shown for the *D1 *gene region, which is represented by the "*D1 *region" box on the consensus assembly and is illustrated in the enlarged regions of the various assemblies. The multi-colored *D1*-g/y gene and gene fragments indicate hybrid genes in which a mix of sequencing reads from *D1*-g and *D1*-y are incorrectly assigned to a single region. The gene fragments marked *D1 *and shown in gray do not contain enough sequence to identify them as *D1*-g or *D1*-y. The primer positions are indicated by number (see Table 2) at the bottom with the forward primers (For) on the top line and the reverse primers (Ref) on the bottom line. Assemblies 3, 6, 11, 12, and 15 have been omitted because of assembly errors based on incorrect positioning of primers.

A variety of parameters were compared among the 12 assemblies in addition to the BCM-HGSC assembly (Figure 2). Sizes of the assembled sequences ranged from 110,951 - 120,165 nt in two to five unordered, unoriented scaffolds (Table 3). Each assembly consisted of a large scaffold of 94,782 - 109,918 nt (82 to 99% of an assembly) and one to four small scaffolds ranging in size from 1,033 - 16,978 nt (Figure 2). Assemblies with two scaffolds (5, 6, 8, and 9) were ordered and oriented based on the vector sequence. Assemblies consisting of three or more scaffolds were ordered and oriented by comparison to the single contig of the BCM-HGSC assembly. While the large scaffolds from each of the assemblies were nearly identical, the smaller scaffolds contained the *Sp185/333 *genes and varied significantly (Figure 2).

All 12 assemblies had three *Sp185/333 *genes that were identical among the assemblies: one with an *A2 *element pattern, one with a *B8 *element pattern and one with an *E2 *element pattern (Figures 1, 2). In addition, each assembly had between one and three fully assembled *D1 *genes, plus most assemblies showed a fragmented or poorly assembled *D1 *gene (Table 3). The sequences of the *D1 *genes varied among the assemblies (shown as yellow and green in Figure 2; see below). In each assembly, the gaps between the small scaffolds were flanked by the *D1 *genes, which indicated that these genes were the source of the conflicts. Varying the unitigger rates altered the number and placement of the *D1 *genes, indicating that further analysis was necessary to obtain the accurate sequence of the *Sp185/333 *gene cluster. For clarity, the *D1 *genes and fragments were given extended names according to their 5' to 3' order within assembly 9: *D1 *yellow (*D1*-y), *D1 *green (*D1*-g), and *D1 *blue (*D1*-b) (Figure 2).

### Experimental validation of the assembled 7096 sequence

A two-fold approach was undertaken to validate the assemblies experimentally. First, PFGE and PCR were used to determine the size of the BAC insert and to confirm the existence and size of the three *Sp185/333 *genes present in all assemblies (Figures 1, 2). Second, the region harboring multiple *D1 *genes was analyzed more closely using PCR, cloning, sequencing, and restriction enzyme analysis. These results were used to reject incorrect assemblies, including the assembly generated by BCM-HGSC, and ultimately to define the correct 7096 sequence thereby enabling analysis of the *Sp185/333 *gene cluster. The 7096 insert size was estimated to be 117.6 kb by PFGE (data not shown), eliminating assemblies 2, 4, and 6 from further consideration as they were too short (Table 3). qPCR estimation of the *Sp185/333 *gene copy number indicated that there were 5.8 to 6.1 *Sp185/333 *genes present (data not shown), which was in agreement with all of the assemblies, if whole genes plus fragments were considered. The remaining nine assemblies were evaluated in more detail.

**Figure 3:**
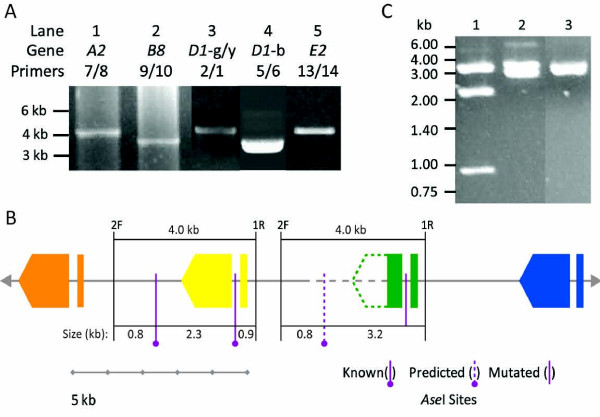
**Experimental evidence supports assembly 9**. **A.** PCR amplification confirms the sizes of the regions surrounding the *A2*, *B8*, *D1*-b, and *E2 *genes. Amplicons in lanes 1 (~4 kb), 2 (~3.6 kb), 4 (~3.6 kb), and 5 (~4 kb), correspond to the sizes of the *A2, B8, D1-*b, and *E2 *genes plus their flanking regions according to sizes predicted in all the candidate assemblies (see Figure 2). A single amplicon of ~4 kb (lane 3) was generated from primers predicted to amplify each D1-g, and D1-y genes plus flanking regions. See Table 2 and Figure 2 for primer information.** B**. Diagram of a region of assembly 9 showing the *D1 *genes (*B8*, orange; see also Figure 2). The subcloned regions of 7096 containing *D1 *genes (amplified with primers 2F and 1R; see Table 2 and Figure 2) are indicated. The assembled sequence for these subclones contains either one (*D1*-y) or two (*D1*-g) *Ase*I restriction sites (purple lines). One of the *Ase*I sites in the *D1*-g gene is mutated by a SNP. Because of the gap in assembly 9, which includes this region (dashed line), one of the *Ase*I sites is predicted (dashed purple line) based on sequence similarity with the *D1*-y subclone. **C**. A SNP obliterates an *Ase*I restriction site and differentiates *D1*-y and *D1*-g genes. PCR amplicons using 2F and 1R primers produce 4 kB fragments. When digested with *Ase*I the clones containing a *D1*-y gene could be differentiated from those with a *D1*-g gene. Lane 1, *D1*-y gene (4.2 kb, 2.3 kb, and 0.9 kb). Lane 2, *D1*-g gene (4.2 kb and 3.2 kb). Lane 3, vector without insert has one *Ase*I site (4 kb).

PCR was used to confirm the sizes of the non-*D1 *genes and to validate the region that included the *D1 *genes, which varied among assemblies (Figure 2). Assembly 9 (Table 3; Figure 2) was chosen as the reference sequence for primer design because it consisted of only two scaffolds and was the second largest assembly (117 kb), suggesting that no genes had been collapsed or duplicated. Primers were designed to flank each of the genes and gene fragments, and PCR was used to confirm the sizes of the genes and their flanking regions. Sizes of the amplified regions surrounding the *A2, B8*, and *E2 *genes were consistent with the predicted sizes from all assemblies (Figure 3A), suggesting that these sequences were likely correct. PCR was also used to resolve the assembly of the *D1 *region. When primers 6F and 5R (Table 2) were used to amplify the *D1*-b gene plus flanking regions, an amplicon of ~3.6 kb was obtained (Figure 3A). This size was different from that predicted in assemblies 11, 12, and 15, eliminating them from further analysis. Primers 2F and 1R (Table 2) annealed in two locations: flanking both the *D1*-y and *D1*-g genes (Figures 2, 3B), which included the gap between the two scaffolds. However, a single amplicon of ~4 kb (Figure 3A) suggested that there was a single *D1 *gene, either *D1*-y or *D1*-g, although it did not rule out the possibility that both *D1*-y and *D1*-g genes were present and that the 2F and 1R primers amplified two fragments of the same size. To resolve the *D1 *gene region, amplicons with *D1*-y or *D1*-g genes using the 2F and 1R primers were cloned (Table 2; Figure 3B). Each 2F/1R subclone had a 4 kb insert with a single *D1 *gene plus a ~200 bp 5' flanking region and a ~2 kb 3' flanking region. To differentiate between the 2F/1R subclones with either the *D1*-g or the *D1*-y gene, the sequences from the assemblies still under consideration (5, 7, 8, 9, 10) plus that from BCM-HGSC were inspected more closely. The Celera assembler did not fully assemble *D1*-g gene and the fragment varied in length from 30 to 840 nt in different assemblies, whereas the BCM-HGSC assembly positioned the *D1*-y gene to the 3' side of a *D1 *gene that was a mix of nucleotides from both *D1*-g and *D1*-y (Figure 2). Based on the sequence of assembly 9, the *D1*-g fragment and the corresponding region of *D1*-y were 99.8% identical over 840 nt with only two SNPs. An *Ase*I site (ATTAAT) in the *D1*-y intron was obliterated in the *D1*-g intron by a SNP (ATTAAC) (Figure 3B). This SNP was confirmed by digestion of the subclones (Figure 3C) such that two patterns of bands were observed. This was consistent with the presence of two different *D1 *genes (*D1-*y and *D1-*g) in addition to *D1-*blue and the sequence from assembly 9.

**Figure 4:**
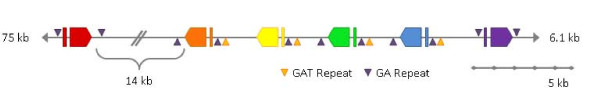
**The experimentally validated assembly of 7096 contains six *Sp185/333 *genes**. The finished assembly and sequence of the region in 7096 containing the *Sp185/333 *genes is shown after experimental confirmation by PCR, PFGE, *Ase*I digests, and sequencing subclones. The BAC contains six *Sp185/333 *genes: one *A2 *gene, one *B8 *gene, three *D1 *genes, and one *E2 *gene (see Figure 1 for element pattern information). All are located at the 3' end of the BAC insert. Gene orientations are indicated and spacing is to scale unless otherwise noted. GA microsatellites are shown flanking each gene and GAT microsatellites are shown to the 5' side of *B8 *and the three *D1 *genes.

To complete the sequence of the *D1*-g gene, which was missing at least part of the 3' end in most of the assemblies, a 2F/1R subclone containing the *D1*-g gene was sequenced at 5.8X coverage with gene specific primers previously designed for sequencing *Sp185/333 *transcripts and cloned genes (Table 2, see also [15,19]). These results showed that assembly 7 and that generated by BCM-HGSC did not have a correct *D1*-g gene and were eliminated from further analysis. The 5' end of the *D1*-y gene, which was a region that varied among assemblies, was also sequenced at 2.94× coverage with gene specific primers (Table 1) and results for a correct *D1*-y gene were not consistent with assemblies 8 and 10, which were also eliminated. These results showed that the *D1*-g gene shared an average 99.7% similarity with *D1*-y and *D1-*b, giving insight into the difficulties for assembling this region of the BAC. The *D1 *genes from assemblies 5 and 9 had 100% identity with the experimentally confirmed *D1*-y and *D1*-g sequences. These two assemblies were nearly identical. The 2 kb gap between the two scaffolds was filled by sequencing (1.97× coverage) a 2F/1R clone containing the *D1*-g gene that spanned the gap. The resulting sequence connected the two scaffolds to complete a final assembly (Figure 4).

### Analysis of the assembled 7096 sequence

#### *Sp185/333 *genes on 7096

The 7096 assembly contained six *Sp185/333 *genes with the following element patterns: one *A2*γ, one *B8*β, one *E2*δ, and three *D1*α genes (Figure 1; Greek letters represent the intron class based on sequence variations; see [15]). The genes varied in size from 1286 to 1881 nt and were of identical structure to that reported previously: two exons and one intron [15,19]. The genes were located within a 34 kb region at the 3' end of the assembled insert (Figure 4) with the *A2 *gene separated from the rest by 14 kb. The remaining five genes were clustered within 20 kb, with intergenic regions of 3.2 ± 0.2 kb. The three *D1 *genes and the *B8 *gene were adjacent to one another in the middle of the cluster and were all oriented in the same direction, whereas the genes at the edges of the cluster, *A2 *and *E2*, were oriented in the opposite direction (Figure 4).

The assembled BAC sequence surrounding the *Sp185/333 *genes was investigated for the basic signatures of transcriptional control, including the TATA box, and polyadenylation signal. In five of the six *Sp185/333 *genes a TATAAA sequence was located 106 nt 5' of the start codon, however, there was a TATACA sequence in same position for the *D1*-g gene. A polyadenylation signal (AATAAA) was identified 175 to 267 nt 3' of the stop codon in four of the six genes. The *D1*-b and *D1*-g genes had a SNP that altered their polyadenylation sequences to ATTAAA and AATATA, respectively. Both the TATAA box and the polyadenylation site for the *D1*-g gene were non-canonical sequences, however the effect of these sequence variations on expression is unknown.

#### *Sp185/333 *sequence diversity

**Figure 5:**
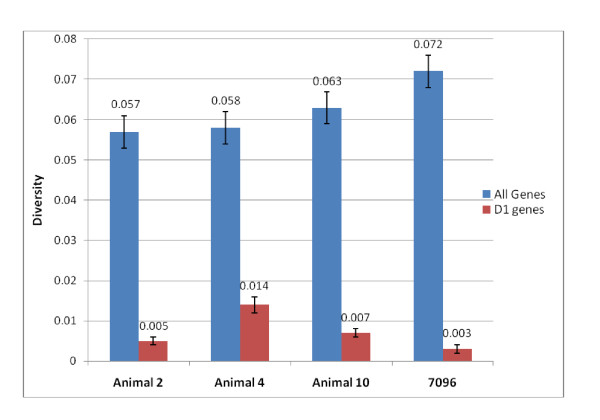
**The *Sp185/333 *genes from 7096 are equally diverse as those randomly isolated from three animals**. Mean pairwise diversity scores of the six *Sp185/333 *genes from 7096 are compared to other *Sp185/333 *genes previously isolated from three other sea urchins (blue bars; 29 genes from animal 4, 87 genes from animal 2, and 49 genes from animal 10 [15]). The *D1 *genes (9 genes from animal 4, 20 genes from animal 2, 6 genes from animal 10, and 3 genes from 7096) were analyzed separately (red bars).

To understand the relationships among the clustered *Sp185/333 *genes, their pairwise sequence diversity was calculated [37] using pairwise gap deletion, which removes positions in which one of the sequences has a gap, to account for variations in element pattern. The mean diversity among the six *Sp185/333 *genes was 0.072 (Figure 5). The *A2 *gene was the most divergent relative to the other *Sp185/333 *genes on the BAC, whereas the *D1 *genes were almost identical (Table 4). The introns were generally more diverse although the introns from the clustered *D1 *genes were highly similar.

**Table 4:** Pairwise diversity of the *Sp185/333 *genes^1^

		*D1-*y	*D1-*g	*D1-*b	*B8*	*A2*
	*D1-*g	0.004				
Full-length gene	*D1-*b	0.003	0.003			
	*B8*	0.071	0.072	0.071		
	*A2*	0.103	0.103	0.103	0.103	
	*E2*	0.087	0.088	0.087	0.082	0.096
						
Exons	*D1-*g	0.004				
	*D1-*b	0.004	0.003			
	*B8*	0.060	0.061	0.060		
	*A2*	0.081	0.082	0.081	0.077	
	*E2*	0.057	0.059	0.057	0.054	0.078
						
Intron	*D1-*g	0.002				
	*D1-*b	0.002	0.005			
	*B8*	0.101	0.103	0.101		
	*A2*	0.168	0.165	0.168	0.175	
	*E2*	0.172	0.176	0.172	0.166	0.139

^1^Diversity scores were generated using MEGA [37] with pairwise comparisons of full-length sequences, exon 1 plus exon 2 without the intron, and the intron alone.

**Figure 6:**
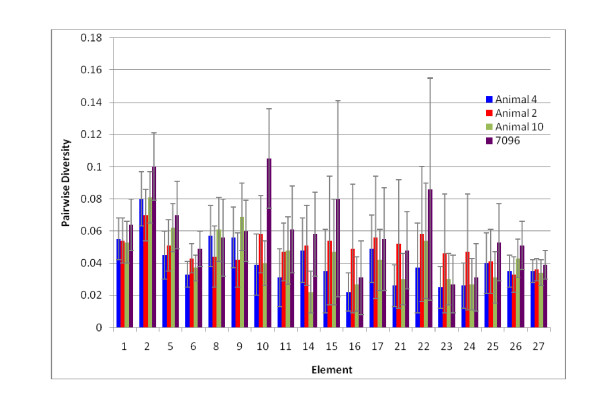
**The element sequence diversity for the *Sp185/333 *genes clustered on 7096 is not different from the element diversity for the *Sp185/333 *genes with unknown genomic organization**. Unique *Sp185/333 *genes from three sea urchins previously isolated (see legend for Figure 5, [15]) and the six genes from 7096 were aligned according to the repeat-based alignment (as in Figure 1, [15]). Genes from individual animals and those from the BAC were used to calculate pairwise diversity scores for each element in MEGA [37]. Average diversity scores and the standard deviations are shown. Elements that were not present in a majority of the sequences were omitted from the analysis.

The diversity of the six clustered *Sp185/333 *genes were compared to 121 unique *Sp185/333 *genes collected randomly from three individual sea urchins for which the relative genomic organization was unknown [15]. The clustered genes on 7096 were slightly more diverse (mean diversity score of 0.072) than genes isolated from the three animals (diversity scores of 0.057 to 0.063) (Figure 5). Because the diversity analysis is influenced by element pattern, and previous data suggested that genes and mRNAs with the same element patterns have nucleotide sequences that are more similar than sequences that do not share element patterns [15,19], the diversity scores were calculated for the *D1 *genes from each of the four sources (three animals and 7096). The three clustered *D1 *genes were slightly more similar to each other than to *D1 *genes isolated from different animals (mean diversity of 0.003), but the differences were not significant (Figure 5). This result was unexpected, given the possible effects of homogenizing forces (e.g. unequal crossing over and gene conversion) and led us to evaluate the distribution of specific element sequences among the genes. Individual elements of the clustered genes were investigated to determine whether they were more likely to share elements with identical sequence compared to elements from genes randomly isolated from other sea urchins. Results indicated that there was no correlation between shared element sequences and tight clustering of the genes (Figure 6).

#### Conserved flanking regions

**Figure 7:**
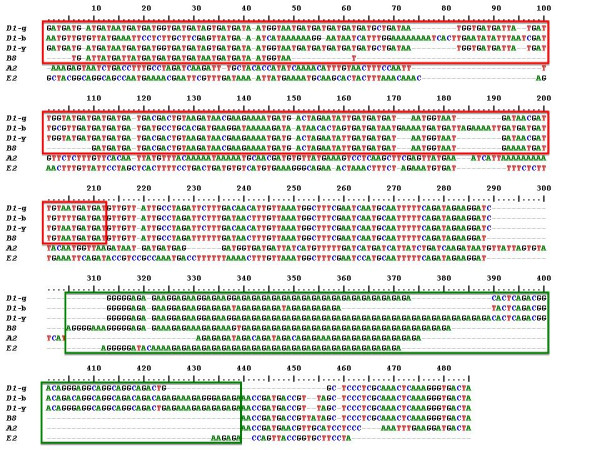
**Alignment of sequences surrounding the GAT and GA microsatellites located to the 5' side of most of the *Sp185/333 *genes**. A region that is approximately 240 to 700 nucleotides 5' of each gene is shown. A GAT microsatellite is not present 5' of the *A2 *and *E2 *genes. The red boxes indicate the GAT microsatellite sequences; the green boxes indicate the GA microsatellite sequences. Dots indicate identity with respect to the sequence on the first line. The alignment was produced using MEGA [37] and edited by hand.

Each of the *Sp185/333 *genes on 7096 was flanked by GA microsatellites (Figure 4). The GA microsatellite positioned on the 5' side of each gene was located ~430 nt from the start codon and ranged in size from 30 - 60 repeats (Figure 7). The GA microsatellite on the 3' side of each gene had 140 - 165 repeats and was located ~300 - 350 nt from the stop codon, except for *A2*, in which the GA microsatellite was ~700 nt 3' of the stop codon. GAT microsatellites, with ~37 - 60 repeats were located ~550 - ~600 nt 5' of the start codon of *B8*, *D1*-g, *D1*-b, and *D1*-y (Figure 4). In general, each gene was flanked by GA microsatellites and a subset of the genes had 5' GAT microsatellites.

**Figure 8:**
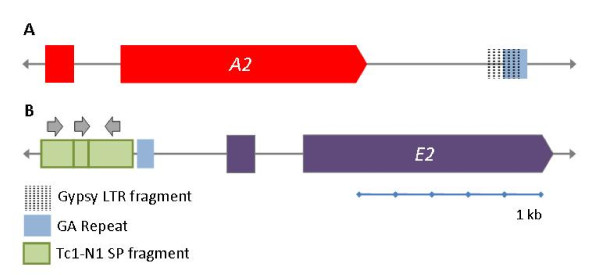
**Transposon fragments are present in the flanking regions for the *A2 *and *E2 *genes**. **A.** Gypsy10-long terminal repeat (LTR)_S fragment. The LTR [GenBank:AAGJ02039135.1] is represented by 139 nt of 2430 nt of the consensus sequence. It is present 684 nt to the 3' side of *A2 *and continues 87 nt into the GA microsatellite. **B.** Three Tc1-N1 SP transposon fragments. Transposon fragments [44] (267 nt, 75 nt, and 140 nt of 553 nt consensus sequence) are present in tandem and positioned 453 nt 5' of *E2 *and 70 nt 5' of the GA microsatellite. Fragment orientation is indicated with arrows.

In addition to microsatellites, fragments of transposable elements were detected among the clustered *Sp185/333 *genes and were associated with two of the GA microsatellites. A portion (139 nt; 5.9%) of a Gypsy10-long terminal repeat (LTR)_S LTR element [GenBank: AAGJ02039135.1] was positioned 684 nt 3' of the *A2 *gene in a region between the gene and the flanking 3' GA microsatellite (Figure 8). It was 50 nt to the 5' side of, and extended 90 nt into the GA repeat, constituting about half of the repeat. Three tandem, incomplete Tc1-N1_SP DNA transposon elements [44], representing 48%, 13%, and 25% of the Tc1 consensus sequence, were positioned 522 nt 5' of the start codon for the *E2 *gene, and 50 nt upstream of the 5' GA microsatellite (Figure 8). It is not known whether these transposable elements may be involved with diversification of the *Sp185/333 *gene family.

**Figure 9:**
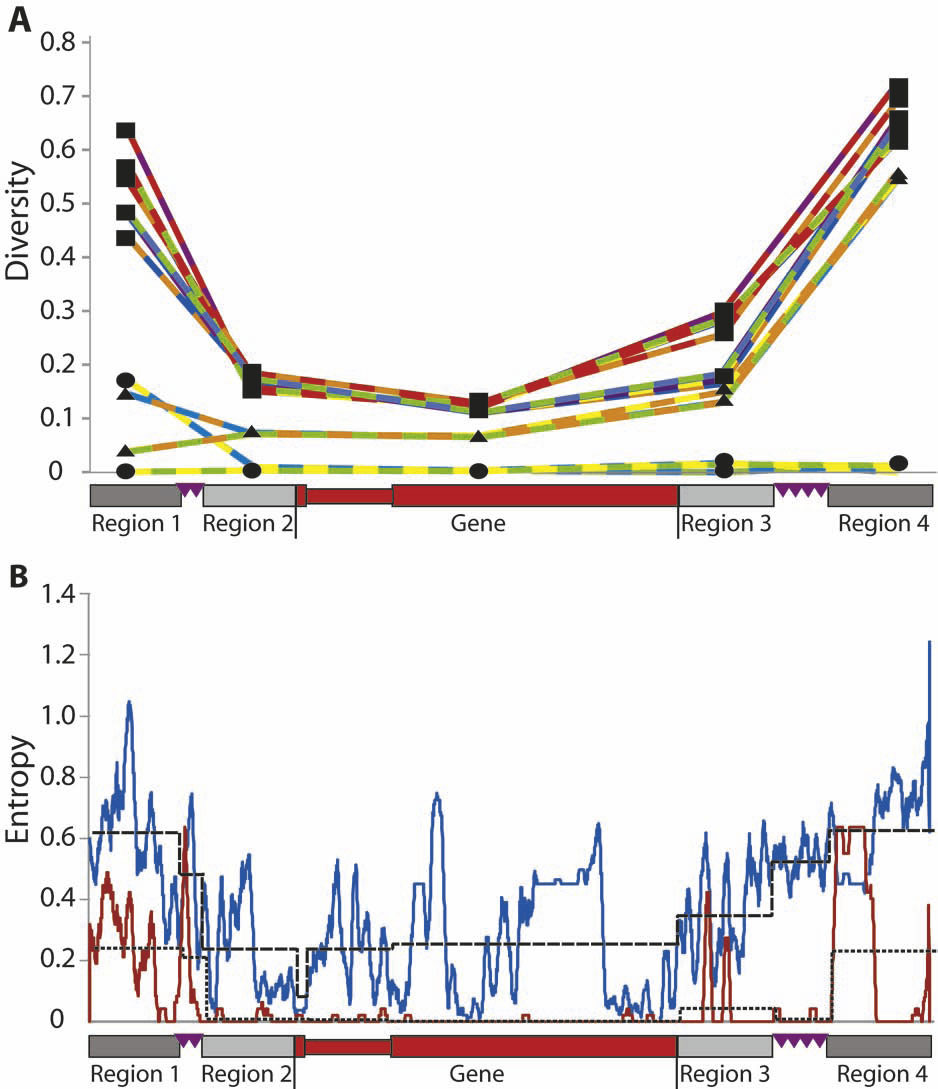
**Microsatellites border the conserved sequence flanking the *Sp185/333 *genes**. **A.** Pairwise diversity of *Sp185/333 *genes and flanking regions. Pairwise diversity was calculated among five regions (gene and four flanking regions) in MEGA [37]. The *Sp185/333 *gene (on the x-axis in red; 5' to 3' orientation) represents a generic gene with the intron shown as a thinner region. The flanking regions are defined by the edge of the gene and the location of the GA microsatellites (purple triangles). Region 1 (~250 nt; dark gray) is upstream of the 5' GA microsatellite. Region 2 (~430 nt; light gray) is between the GA microsatellite and the start codon. Region 3 (~330 nt; light gray) is between the stop codon and the 3' GA microsatellite. Region 4 (~330 nt; dark dray) is downstream of the 3' GA microsatellite. The two colors in each line correspond to the two genes that were used in the pairwise comparison and match the gene colors shown in Figure 4. Three categories of pairwise diversity are i) high (squares) in regions flanking the gene, ii) low (circles) in all regions including the gene sequences, and iii) hybrid (triangles) where pairwise diversity is low in regions 1 and 2 and high in regions 3 and 4. **B**. The microsatellites are boundaries for sequence conservation. Alignments of the genes and flanking sequences were used to calculate the entropy over a 30 nt window that slides 1 nt for each calculation. Entropy scores are shown for the analysis with all six genes (blue line) and for only the three *D1 *genes (red line). The black lines show the average diversity of the regions indicated on the x-axis for all six genes (dashed line), or only the three *D1 *genes (dotted line).

Based on the presence of microsatellites and LTRs and the conserved distances between these repeats relative to the 5' and 3' ends of the genes, the level of sequence conservation was calculated among the genes and among the proximal and distal flanking regions with respect to the GA microsatellites. Genes and flanking regions were divided into five regions: the gene including the intron, the regions between each exon and the respective flanking GA microsatellite (proximal regions 2 and 3 in Figure 9A), and the regions outside of each GA microsatellite (distal regions 1 and 4 in Figure 9A). The pairwise diversity for each of these regions was calculated for all pairs of genes and regions. The microsatellites were not included in the analysis because variations in copy number precluded a robust alignment. Three regions were relatively conserved; the proximal flanking region 2 (between the 5' GA microsatellite and the start codon [average diversity = 0.115; Figure 9A]) and region 3 (between the stop codon and the 3' GA microsatellite [average diversity = 0.164]), and the gene sequences themselves (average diversity = 0.084). These three regions had relatively low diversity scores indicating sequence conservation between the microsatellites including the genes and their proximal flanking regions.

The pairwise diversity scores for the distal regions outside of the flanking GA microsatellites (regions 1 and 4, Figure 9A) defined three categories of gene diversity: high, hybrid, and low. The high diversity category for the distal regions included the pairwise diversity scores between either *A2 *or *E2 *and each of the other genes. Results showed a sharp increase in the sequence diversity between the proximal and distal flanking regions with respect to the GA microsatellites. This indicated that the proximal flanking sequences were generally more similar to each other than the distal flanking regions were to each other. The hybrid diversity category included pairwise comparisons between *B8 *and each of the *D1 *genes with respect to the two distal regions (Figure 9A). There was low diversity in region 1 (average diversity = 0.051) and high diversity in region 4 (average diversity = 0.548). Regions 1 and 2 for the *B8 *gene were conserved with respect to all of the *D1 *genes because that side of *B8 *was adjacent to the GAT microsatellite and part of the intergenic region oriented towards the *D1*-y gene (see Figure 4). On the other hand, regions 3 and 4 of the *B8 *gene had divergent sequence with respect to the corresponding *D1 *gene regions, and were part of the intergenic region oriented towards the *A2 *gene. The *B8 *gene therefore represented an interesting hybrid of conserved and divergent flanking regions. The low diversity category included pairwise comparisons among the three *D1 *genes, which had low scores in all regions (Figure 9A).

The patterns of sequence diversity among the genes and the flanking regions were analyzed more closely by calculating the average diversity (using the entropy equation) over a sliding 30 bp window (Figure 9B). The diversity of all six sequences indicated that the genes, as well as the proximal flanking regions (2 and 3) were relatively conserved, and that the sequences diverged sharply distal to the GA microsatellites (regions 1 and 4). When only the *D1 *genes were analyzed, they showed much greater identity in all regions compared to the result that included all of the genes (Figure 9B). The *D1 *genes were almost identical, with slightly less identity in the proximal flanking regions (2 and 3) and somewhat less identity in the distal flanking regions (1 and 4). In all cases, the microsatellites marked the boundaries between the more conserved and less conserved flanking sequence.

**Figure 10:**
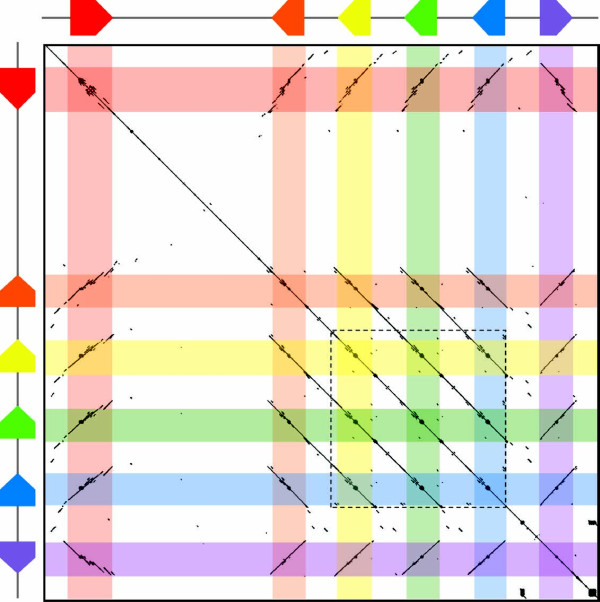
**Dot plot of the *Sp185/333 *gene cluster shows gene and segment duplication**. The *Sp185/333 *gene cluster of 34 kB is plotted against itself. The colored pentagrams indicate the positions of each of the *Sp185/333 *genes. Matching sequence appears as diagonal lines of dots indicating similar sequences are present in the same or opposite orientation. The genes plus a short region upstream of each of the genes are conserved. The dotted box illustrates the region of segmental duplications as indicated by the length and number of parallel, diagonal lines. The total length of the segmental duplications is ~13.7 kb and consists of three segments that each include a *D1 *gene (yellow, green and blue).

The low diversity surrounding the *D1 *genes suggested that conserved sequence may extend beyond the distal flanking regions that were analyzed. A dot plot of the BAC sequence that included the *Sp185/333 *gene cluster was used to determine the extent of conservation in the intergenic regions between all of the genes, and the *D1 *flanking regions in particular (Figure 10). Results were in agreement with the diversity (entropy) scores and showed conserved sequence of the genes and the proximal flanking regions that were bounded by the GA microsatellites. Furthermore, the dot plot also showed large, or segmental, tandem duplications that included each of the *D1 *genes and their intergenic sequences (Figure 10). The segmental duplication was ~13.5 kb in total and consisted of three equal tandem segments each with a single *D1 *gene and its flanking regions (Figure 10). Each duplication included ~700 nt 5' and ~2.3 kb 3' of each *D1 *gene and was bounded by GAT microsatellites. The sequence conservation of the 5' flanking region of the *B8 *gene, noted from the low pairwise diversity scores, appeared to be part of the segmental duplication. However, because the *B8 *gene had a different element pattern from the duplicated *D1 *genes, and because the conserved 3' flanking region of *B8 *only extended 330 nt to the GA microsatellite, we speculate that the putative duplication of the *B8 *gene mediated by the GA microsatellites was adjacent to the segmental duplication that included *D1 *genes but was not part of it. In general, the patterns of sequence conservation and positions of microsatellites suggest multiple mechanisms of sequence duplication and diversification within the *Sp185/333 *gene family.

## Discussion

**Figure 11:**
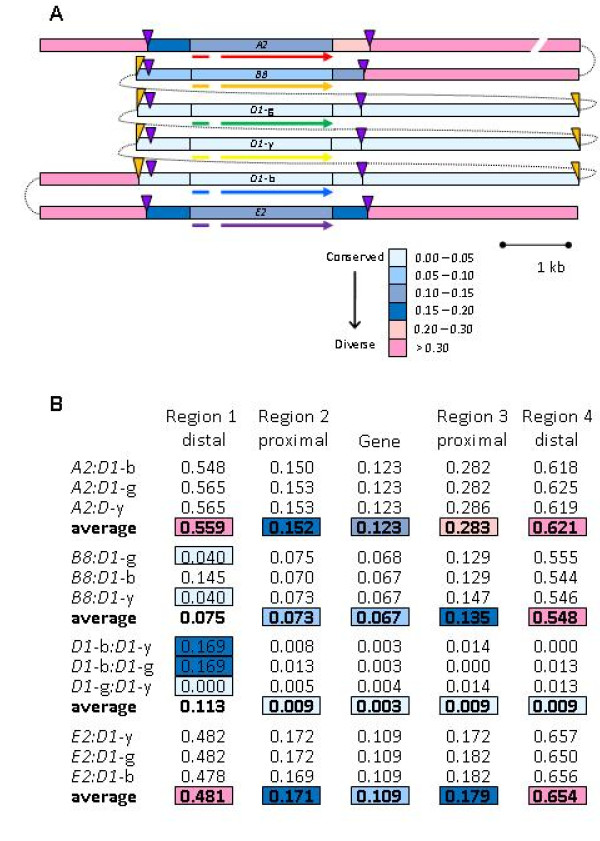
**Duplications of genes and larger segments may be mediated by microsatellites**. **A**. Alignment of the *Sp185/333 *genes and flanking regions. The genes (indicated as colored arrows) and proximal flanking regions between the GA microsatellites (purple triangles) are conserved. (For definitions of distal and proximal flanking regions, see the text and legend for Figure 9.) Conserved sequence between the GAT microsatellites (orange triangles) includes the three *D1 *genes and the associated intergenic regions. The GAT microsatellites are split into half triangles, except for the one located on the 5' side of *D1*-b, to show their positions relative to each gene. All of the intergenic sequence between the genes is shown, except for the region between *A2 *and *B8*. The fine dotted lines indicate how the sequences fit together on the BAC. The legend shows variations in color that relate to ranges of pairwise diversity scores based on results in B. **B**. Pairwise sequence diversity relative to the *D1*-y and *D1*-g genes. The level of sequence conservation is based on pairwise diversity scores for each of the *Sp185/333 *genes compared to the *D1*-y and *D1*-g genes. Colors in the table correlate to colors in the alignment in A.

The data presented here are the first finishing-level sequence of a small cluster of *Sp185/333 *genes on a BAC insert. Multiple assemblies were generated with varying parameters to account for potential gene collapse or artificial duplication/expansion, which is a significant problem for regions with shared sequence or many repeats. The optimal assembly was verified by molecular biology techniques. We describe a unique perspective on sequence assembly and validation, particularly the local adjustment of assembly parameters to account for regions with repeats that are often misassembled when global parameters are used to assemble whole genomes. Six *Sp185/333 *genes are clustered within 34 kb and have an intron/exon structure that is consistent with previous reports [15,19]. Each of the three *D1 *genes is positioned within tandemly duplicated segments that include the intergenic regions and is delineated by GAT microsatellites (Figure 11). We speculate that these microsatellites are involved with this recent duplication event and that the SNPs within segments are due to subsequent sequence diversification. Furthermore, all six genes on the BAC display significant similarity within the coding regions and the 5' and 3' proximal flanking regions, which are bounded by GA microsatellites, suggesting duplications of these shorter regions (Figure 11). There may be multiple mechanisms that operate in this gene cluster and that 1) may employ microsatellites to promote sequence diversification, 2) may also block sequence homogenization of the region resulting from gene conversion, and 3) may prevent the generation of gene fragments and pseudogenes. Together, this would contribute to and maintain the extraordinary diversity of this gene family.

### Microsatellites and sequence diversification

Microsatellites are common in the genomes of most organisms, although their importance in function and evolution has been debated for years [45-47]. Microsatellites have been associated with regions of increased recombination in a number of organisms, including yeast [48] and, to a lesser extent, mammals [49-51]. Microsatellites have also been associated with increased genomic diversity by promoting sequence duplications, gene conversion, crossovers, and generating local recombination hotspots [45,48-50,52,53]. A novel segmental duplication mechanism has been reported wherein duplications are generated by template switching between microsatellites [54] and appear to stimulate recombination in plasmids [55-58]. The sequence diversity observed for the *Sp185/333 *genes may result, in part, from recent and frequent recombination [22]. The combination of gene and segmental duplications in addition to gene recombination may be a powerful system for generating and or maintaining sequence diversity in this gene family.

### Heterogeneous gene clusters

Many large gene families in organisms from plants to mammals have immune related functions. In humans, the major histocompatability complex (MHC) has over 160 genes that diversify through sequence exchange and duplication [59] and clusters of *R *genes in higher plants also maintain diversity through sequence exchange and recombination (reviewed in [11]). The *Sp185/333 *gene family is another example of a large diverse immune related gene family (reviewed in [16]). The *Sp185/333 *cluster on the 7096 BAC is positioned 6.1 kb from the end of the insert, which makes it unclear whether this cluster is one of several small isolated clusters in the genome, or whether it is the end of a large cluster with additional linked genes that might be identified from overlapping BACs. Examples of both large and small clusters of linked genes involved in immune responsiveness have been found in other organisms. The nucleotide binding, leucine-rich repeats (NB-LRR) subclass of *R *genes in *Arabidopsis *has 149 members of which 109 are clustered into small groups consisting of two to eight genes [60,61]. Similarly, the sea urchin Toll-like receptor (TLR) genes are clustered in small groups that are spread throughout the genome [4,62]. Multiple large clusters of over 1,000 variant surface glycoprotein (VSG) genes in *Trypanosoma brucei *are distributed into 15 sizeable (40-60 kb) telomeric sites [63].

The six *Sp185/333 *genes on the 7096 BAC form a heterogeneous cluster with four different element patterns. Except for the *D1 *genes, there is no correlation between proximity and sequence similarity among the linked genes on 7096 compared to genes that have been randomly isolated with unknown linkage (Figure 5). Although we suggest that the genes may be the result of duplications mediated by the GA microsatellites, it does not appear that the different element patterns of the clustered genes on 7096 are the result of tandem gene duplications from a single gene followed by sequence diversification. Consequently, the *Sp185/333 *gene cluster appears as a heterogeneous cluster of genes with different element patterns. Heterogeneous clusters of tandemly linked *R *genes have been investigated in *Arabidopsis *in which more than ten clusters have intermingled genes from two different subfamilies: the Toll/interleukin-1 LRR (TNL) subfamily and the coiled-coil region LRR (CNL) subfamily [60,61]. A proposed advantage of heterogeneous clusters is a block to gene homogenization and maintenance of diversity among the members of the cluster [64]. Two models have been proposed to explain the origins of heterogeneous clusters. The 'rapid rearrangement' model suggests that small areas consisting of one to a few genes are ectopically duplicated such that genes are copied to unlinked regions of the genome [60,61]. The 'conserved synteny' model suggests that large-scale segmental duplications are moved to new genomic locations, including different chromosomes [11,65]. Evidence for these models is based on the level of synteny, or lack thereof, in regions surrounding heterogeneous clusters. It is not clear whether either of these mechanisms functions within the *Sp185/333 *family, however, the notion of copying sequences from within the GA repeats to other locations of the genome with similar GA repeats is consistent with the heterogeneous mixture of *Sp185/333 *genes in the cluster. It is also consistent with a rapid rate of gene diversification as deduced from molecular clock analysis [16] and as proposed for rapid gene recombination [22].

#### Gene conversion

In addition to ectopic duplication of genes and segments to produce heterogeneous clusters, gene conversion may also be involved in sequence diversification, which may be promoted not only by the GA microsatellites, but also by the repeats and shared element sequences within the coding region. Six types of coding region repeats were first reported for ESTs and full length transcripts [17,19] and are present in the second exon in both tandem and mixed interspersed organization (Figure 1) [15]. Within the repeats, shorter, simple repeats are also present [22]. In addition, many of the genes share element sequences and simple repeats, and, on a larger scale, the genes themselves can be viewed as imperfect repeats. If the similarity among the *Sp185/333 *sequences promotes crossovers and gene conversion, these activities would lead to sequence homogenization of the genes, the flanking regions, and possibly an entire region harboring *Sp185/333 *genes. This would be counter-productive for maintaining a diverse gene family with putative immunological functions. However, because sequence similarity among the genes decreases outside of the GA microsatellites, it suggests that regions that undergo sequence exchange are limited to the span between the GA microsatellites. The flanking microsatellites may act to block the progression of DNA strand exchange during crossovers and gene conversion, protecting the entire region from sequence homogenization including nearby *Sp185/333 *genes. An example of this type of result that has been experimentally observed in yeast [53]. Overall, we postulate two activities that may function simultaneously to generate and regulate sequence diversity among the cluster of *Sp185/333 *genes. Both the GA and GAT microsatellites may promote duplication of genes and larger segments leading to diversification perhaps by recombination. On the other hand, the shared sequences within the coding regions may promote an unknown level of gene conversion among both closely linked and unlinked genes that could preserve the heterogeneous nature of the cluster. Furthermore, strand exchange during gene conversion may be restricted to the genes and proximal flanking regions by the GA microsatellites that might block the spread of sequence homogenization to other genes within a tight cluster.

#### Pseudogenes

Gene fragments and pseudogenes are common in clusters of genes belonging to the same family [66,67] and often result from common mechanisms of duplication and diversification such as unequal crossing over and tandem duplication. Surprisingly, no gene fragments have been found in the *Sp185/333 *family even after extensive searches of the genome, and only one pseudogene has been identified (of 171 genes sequenced) that appears to be the result of retrotransposition [15]. The remaining 170 sequenced genes have perfect open reading frames and splice signals. We speculate that the mechanisms that promote a rapid rate of gene diversification, as predicted by Buckley et al. [22] and as proposed above, may be under controls to avoid generating fragmented and non-functional genes. The flanking microsatellites and their putative block to DNA strand exchange may be involved in maintaining the reading frame fidelity while promoting diversification, given their location at the edges of the conserved flanking regions of the genes and at the edges of the tandem segmental duplications.

#### *A2 *Gene Diversity

The *A2 *gene can be categorized as the outlier of the cluster for more than just reasons of distance. It has the highest sequence diversity compared to the other genes within the cluster (Table 4, Figure 11B) and it has variant GA microsatellites. Previous reports show that *large *genes such as *A2 *(large genes always have elements 2 through 5, see Figure 1) are strikingly different from *small *genes (*B, D *and *E *patterns, see Figure 1) that make up the rest of this cluster (see [15]). The sequences of the shared elements are entirely different [22] even though the *large *and *small *genes have a somewhat comparable complement of elements within the patterns (Figure 1). This prompted previous speculation that the *A2 *genes may be spatially separated from the rest of the *Sp185/333 *genes, perhaps located in a separate cluster that would prevent recombination among *large *and *small *genes [22]. Consequently, it was unexpected to find an *A2 *gene clustered near five *Sp185/333 *genes of the *small *category. Differences between the element diversity in the *A2 *gene compared to the other genes in the cluster may be due to its separation from the other genes by 14 kb, however, variations in the 3' flanking GA microsatellite may also be involved, preventing recombination between the *A2 *gene and the other *Sp185/333 *genes within the cluster. If altered GA microsatellites are present in the other *A2 *genes throughout the genome, this may restrict recombination or gene conversion to within the *A *type element pattern category and maintain the sequence diversity for all of the *A *type genes so that they share a similar element pattern and individual element sequences. A possible origin for the variation of the GA microsatellite associated with the *A2 *gene is the LTR element fragments that are interspersed within this particular microsatellite. Whether this unique 3' GA microsatellite is common to all *A2 *genes and to all genes in the *large *category and whether it is involved in maintaining separate element sequences between *large *vs. *small *genes is unknown and will require additional sequence data.

### Duplications imply deletions

We hypothesize above that the recent segmental duplications that include the three *D1 *genes within the cluster may be mediated by the GAT microsatellites. However, the presence of duplications implies that deletions also occur, which are difficult or impossible to detect. Preliminary PCR amplification of *Sp185/333 *sequences on two BACs, 7096 and 181662, indicated that both had *Sp185/333 *genes in different arrangements (data not shown). Initial sequencing of 181662 BAC (completed before 2006) resulted in 15 unordered contigs [116 kb, GenBank:AC181662.1] and included one contig with a complete second exon from a *Sp185/333 *gene with an open reading frame and a 3' flanking GA microsatellite. In 2008, a finishing-level sequence for 181662 (136.6 kb) resulted in a single contig with no *Sp185/333 *genes, although GA microsatellites were present. Intergenic distances between GA microsatellites that flank the *Sp185/333 *genes on 7096 range from 1.9 to 2.5 kb, although the spacing between *B8 *and *A2 *is much larger. The distances between large GA microsatellites (similar in repeat numbers to those surrounding the *Sp185/333 *genes reported here) on 181662 are 1.3, 1.4 and 2.6 kb. This spacing is typical for the majority of the *Sp185/333 *genes as assayed by intergenic PCR amplification of genomic DNA [15]. We speculate that if the GA microsatellites mediate gene deletion and that this occurred during propagation of the BAC in culture, then the positions of the microsatellites on 181662 suggest that the *Sp185/333 *genes were spaced apart from each other similar to that for 7096. In comparison, results from another BAC, 076N15 (139 kb; see http://www.spbase.org/SpBase/resources/bac_sequences.php for BAC sequence), that harbors homologues of two complement genes and does not have *Sp1865/333 *genes, has six large GA microsatellites that are spaced apart by 4 - 33.8 kb. This spacing is much greater than reported here for either 7096 or 181662. Although it is not known whether the deletion of *Sp185/333 *genes on 181662 was based on instability from the GA microsatellites, it is intriguing that these microsatellites may mediate gene both duplication and deletion.

### Gene copy number does not correlate with the level of gene expression

Of the four different element patterns present in the genes within the cluster, two are of particular interest because of differences in both gene copy numbers and expression levels. The presence of three *D1 *genes vs. single copies of genes with other element patterns is consistent with the previous observation that *D1 *is the most commonly observed element pattern among genes [15]. Yet despite the higher frequency, expression of *D1 *genes is relatively low compared to expression of *E2 *genes [18,19]. Based on the cluster of genes reported here, reduced expression may be the result of a non-consensus TATA box associated with the *D1*-g gene and non-consensus polyadenylation sites associated with the *D1*-g and *D1*-b genes. This raises the possibility that these genes may either be expressed less efficiently or they may be pseudogenes; however, it is not known whether other *D1 *genes in the genome also have variant TATA box and polyadenylation sites. On the other hand, the *E2 *gene, which is most commonly expressed [18,19], is observed less often in randomly sequenced genes [15] and is present as a single copy in the sequenced cluster. This suggests that increased expression of *E2 *gene(s) in the genome may be the result of very active promoters that overcome an estimated lower gene copy number relative to *D1 *genes (12-18 *E2 *genes vs. 30-45 *D1 *genes [68], KM Buckley, unpublished). It is important to note however, that although *E2 *is the most commonly isolated element pattern among transcripts in response to immune challenge, a limited number of pathogen-associated molecular patterns (PAMPs) have been tested for the induction of *Sp185/333 *expression [17,18,20]. Testing additional PAMPs may show a variety of response levels for *Sp185/333 *genes with different element patterns that are present in the genome at different frequencies. Furthermore, the disparity in expression levels for genes with different element patterns may suggest that expression of each gene may be independently controlled by *cis *regulatory elements as opposed to a group expression control mechanism. This hypothesis is supported by comparisons between sequences from genes and messages for three sea urchins which shows that most of the messages (59% to 93% for different individuals) are likely transcribed from a single gene per animal [68].

## Conclusions

### Conclusions: Diversification of the *Sp185/333 *gene family

Previous studies of the *Sp185/333 *gene family and encoded proteins have provided evidence of several different mechanisms that ultimately diversify the pool of Sp185/333 proteins: gene recombination [22], RNA editing [68], and post-translational modifications [21,69]. To this body of data, we present a computational basis for postulating three additional diversification mechanisms; i) gene and segmental duplications driven by sequence similarities among the genes and the flanking microsatellites, ii) ectopic duplication, and iii) gene conversion promoted by coding region sequence similarities with strand exchange blocked by flanking microsatellites. Additional mechanisms for generating sequence diversity in the *Sp185/333 *gene family are undoubtedly possible. The *Sp185/333 *gene family in the purple sea urchin remains an interesting example of a complex invertebrate immune system that functions effectively in host protection against the myriad of possible pathogens in the marine environment.

## Abbreviations

BAC: bacterial artificial chromosome; BCM-HGSC: Baylor College of Medicine - Human Genome Sequencing Center; CNL: coiled-coil region LRR; dNTP: deoxynucleotide triphosphate; DTCS: dye terminator cycle sequencing; FRePs: fibrinogen related proteins; gDNA: genomic DNA; LTR: long terminal repeat; MHC: major histocompatability complex; NB-LRR: nucleotide binding, leucine-rich repeats; PAMPs: pathogen-associated molecular patterns; PFG: pulsed-field gel; PFGE: pulsed-field gel electrophoresis; qPCR: quantitative polymerase chain reaction; SLS: sample loading solution; TBE: Tris borate EDTA; TLR: Toll-like receptor; TNL: Toll/interleukin-1 LRR; VCBPs: variable region-containing chitin-binding proteins; VSG: variant surface glycoprotein; WGS: whole genome shotgun;

## Authors' contributions

CAM and KMB carried out the computational and bench work. CAM wrote the first version of the mansucript. KMB edited the final version of the manuscript and revised the figures. RLE made subclones of the genes and intergenic regions from the BAC and directed the undergraduate students in the lab. LCS oversaw the research, generated the funding, and edited the manuscript and figures. All authors read and approved the final manuscript.

## Author Information

Current positions:

CAM is a doctoral student in the Department of Biology, Boston College, Boston, MA. KMB is a postdoctoral researcher in the Department of Immunology, Sunnybrook Research Institute, University of Toronto, Toronto, ON, Canada. RLE is a research scientist at TECHLAB Inc. in Blacksburg, VA. LCS is a Professor of Biology at George Washington University, Washington DC.
